# The Relationship between Left Atrial Volume and Ventricular Arrhythmias in the Patients with Dilated Cardiomyopathy

**Published:** 2014-01-01

**Authors:** Abdullah Kaplan, Ahmet Gurdal, Cansu Akdeniz, Omer Kiraslan, Ahmet K. Bilge

**Affiliations:** 1 Department of Cardiology, Istanbul Faculty of Medicine, Istanbul University, Istanbul, Turkey

**Keywords:** Left Ventricular Dysfunction, Implantable Defibrillator, Arrhythmia

## Abstract

**Background:**

The present study aimed to investigate the relationship between Left Atrial Volume (LAV), a marker of diastolic dysfunction, and the frequency of malignant ventricular arrhythmia in the patients with left ventricular dysfunction and a previously implanted Implantable Cardioverter Defibrillator (ICD) device.

**Methods:**

This cross-sectional study was conducted on 32 patients with ischemic or idiopathic dilated cardiomyopathy, each having had an ICD device implanted at least 1 year beforehand. The ventricular arrhythmia episodes which were detected and stored by the device were retrieved and evaluated. In addition to routine echocardiographic measurements, all the patients had their LAV and LAV indexes calculated.

After all, student’s t-test, Mann-Whitney U test, and Pearson correlation were used to analyze the data. Besides, P value < 0.05 was considered as statistically significant.

**Results:**

This study was conducted on 4 female and 28 male patients with the mean age of 58.41 ± 9.97 years. Among the study patients, 21 had at least one previous myocardial infarction. In addition, 17 patients had experienced sustained VT or VF within the last year. No significant difference was found between the patients with and without malignant ventricular arrhythmias (sustained VT or VF) regarding LAV (17 patients with arrhythmia (68 + 23.39 mL) vs. 15 patients without arrhythmia (55.13 ± 20.41 mL); P = 0.100). However, the LAV index was significantly higher in the patients with arrhythmia compared to those without arrhythmia (39.27 ± 12.19 mL / m2 vs. 25.18 ± 7.45 mL / m2; P = 0.004).

Both LAV (73.33 ± 17.64 mL and 57.52 ± 23.15 mL, respectively; P = 0.040) and LAV index (40.86 ± 8.47 mL / m2 and 28.20 ± 11.77 mL / m2, respectively; P = 0.010) were significantly greater in the patients with ICD shock therapy within the last year compared to the others. However, both groups were similar regarding Left Ventricular Volume (LVV), LVV index, and ejection fraction.

**Conclusions:**

The study findings demonstrated that LAV and LAV index could be used in detecting the patients who are at high risk of malignant ventricular arrhythmias.

## 1. Background

Heart failure and serious ventricular arrhythmias are general findings of Left Ventricular (LV) dysfunction. Yet, these problems may also arise in the absence of such dysfunction (1). Ventricular arrhythmia and LV dysfunction are independently associated with mortality risk in the patients with previous myocardial infarction (2-5). After a severe fall in Ejection Fraction (EF) the frequency of ventricular arrhythmia has been found to be associated with LV size rather than EF (6).

Many studies have thoroughly investigated the association between systolic dysfunction and ventricular arrhythmia and death. Nonetheless, the importance of diastolic dysfunction has not been clarified in the patients with both systolic dysfunction and fatal ventricular arrhythmias.

Left Atrial Volume (LAV) is a marker of diastolic dysfunction in the patients without Atrial Fibrillation or valvular heart disease (7). A previous study revealed a strong association between LAV and diastolic function grade (8). The present study aims to investigate the association between LAV reflecting diastolic dysfunction grade and the frequency of ventricular arrhythmia in the patients with LV dysfunction. In fact, we intended to unravel the association between LAV reflecting chronic diastolic dysfunction grade and malign ventricular arrhythmias detected by ICD devices within the last year.

## 2. Materials and Methods

The present cross-sectional study was carried out on 32 patients at the Istanbul University Cardiology Clinic. The data were collected from the patients who had been consecutively admitted to our outpatient clinic. The inclusion criterion of the study was being an ischemic or idiopathic dilated CMP patient having had an ICD implanted at least 1 year beforehand. On the other hand, the exclusion criteria of the study were LV aneurysm, biventricular ICD, hypertrophic or restrictive CMP, severe mitral insufficiency, previous valve replacement, rheumatic valve disease, and atrial fibrillation.

The study was approved by the Ethics Committee of Istanbul Faculty of Medicine, Istanbul University.

All the patients’ physical examinations had been done before ICD interrogation and echocardiographic measurements. Moreover, the patients’ functional capacity was determined according to the classification of New York Heart Association (NYHA).

All the patients had ICD interrogation twice a year and the malign arrhythmias which had been stored in the memory of ICD device were examined. Overall, there were 2 types of ICDs in the patients: Guidant (St. Paul, MN, USA) and Medtronic (Medtronic Inc. Mineapolis, MN, USA). Ventricular arrhythmia episodes stored in the ICDs were retrieved. Then, both true and false arrhythmias, either terminated by shock or ATP therapy, were differentiated from the intracardiac electrocardiographic records (EGM) of the device. Only true arrhythmias were taken into consideration, while inappropriate shock and ATP therapies as well as non-sustained VT episodes were excluded.

It should be mentioned that the physicians who did the echocardiographic examinations were unaware of the ICD records. All the patients underwent a 2-dimensional, “pulsed wave” Doppler and a tissue Doppler echocradiography with standard records. All the measurements were made by means of Vivid 7 (GE Medical Systems). In addition, LV end-systolic and end-diastolic volumes as well as EFs were measured through biplane Simpson method. Moreover, mitral flow velocities and mitral annular velocities were measured using “pulsed wave” Doppler and tissue Doppler techniques at the apical four chamber window. For mitral flow velocity, the “sample volume” was placed on the tips of the mitral leaflets and early (E) and late (A) peak filling velocities of LV were measured for tissue Doppler measurement. On the other hand, the “sample volume” was placed on the mitral valve lateral and septal annuli and mitral annulus systolic (S’), early diastolic (E), and late diastolic (A) velocities were measured.

LAV was calculated by inserting the LA diameters measured at the parasternal long axis and apical four chamber views into the following formula: LAV = D1xD2xD3x 0.523 (26). At the parasternal long axis, the first D1 was measured at end-systole. End-systolic horizontal diameter of LA (D2) at the apical four chamber view and the anteroposterior diameter (D3) formed the second and the third measurements, respectively. LA and LV volume indexes were obtained by dividing the echocardiographically measured volumes by the calculated body surface area. Besides, body surface area was calculated by placing height (cm) and weight (kg) into the DuBois and DuBois Formula: BSA = (W 0.425 x H 0.725) x 0.007184.

### 2.1. Statistical Analysis

The data were presented with descriptive statistics as frequencies and means ± standard deviation. All the statistical analyses were performed using the SPSS statistical software, V10.0 (Statistical Package for the Social Sciences INC., Chicago, USA). Differences in normally distributed continuous variables were assessed by independent Student’s t-test. In addition, the categorical variables were compared through chi-square test. Mann-Whitney U test was also used to compare the non-normally distributed continuous variables. Moreover, Pearson’s correlation coefficient was used to determine the correlations between arrhythmic episodes and clinical as well as echocardiographic parameters. P value < 0.05 was considered as statistically significant.

## 3. Results

The present study was conducted on 32 (female: 4, male: 28) patients with the mean age of 58.41 ± 9.97 years (38 – 79 years). Among the study patients, 21 had at least one previous myocardial infarction. The results revealed no significant differences among age, LAV, LVV, volume indexes, EF, Doppler, and tissue Doppler parameters with regard to CMP etiology. The mean EF was 31 ± 5.45% (20 - 48%), with only one patient having an EF of above 40%. Echocardiographic, demographic, and ICD arrhythmia interrogation features are presented in Tables 1-3. 

**Table 1.** Basic Demographic Characteristics of the Patients[Table-fn fn7182]

	Patient Number	Percent
**Coronary artery disease**	21	65.6
**Normal coronary arteries**	11	34.3
**Antiarrhythmic medication**	17	53.1
**Amiadorone**	15	46.8
**Propofenone**	1	03.1
**Sotalol**	1	03.1
**Amiadorone + Propofenone**	1	03.1
**ACE inhibitor**	22	68.7
**ARB**	5	15.6
**Beta blocker**	28	87.5
**Sipirinolactone**	15	46.8
**Furosemide**	25	78.1
**Digoxin**	3	09.4
**NYHA FC I**	17	53.1
**NYHA FC II**	12	37.5
**NYHA FC III**	3	09.4

Abbreviations: ACE, Angiotensin Converting Enzyme; ARB, Angiotensin Receptor Blocker; NYHA FC, New York Heart Association Functional Classification

**Table 2.** Mean Values of the Echocardiographic Findings of the Patients[Table-fn fn7183]

Echocardiographic Findings	Mean ± SD (rang)
**LAV (mL)**	61.97 ± 22.65 (32 - 116)
**LAV index (mL / m^2^)**	32.23 ± 12.22 (16 - 58)
**LVV (mL)**	171.62 ± 56.54 (84 - 275)
**LVV index (mL / m^2^)**	86.67 ± 29.97 (38 - 130)
**LAV / LVV**	39.76 ± 15.35 (15 - 89)
**EF (%)**	31 ± 5.45 (20 - 48)

Abbreviations: LAV, Left Atrial Volume; LVV, Left Ventricular Volume; EF, Ejection Fraction

**Table 3.** The Number of the Patients Who Had Experienced Ventricular Arrhythmias and Administered Therapies Stored by the ICD Device within the Last Year[Table-fn fn7184]

	Number of Patients ^*^	Percent
**VF**	2	06.3
**Sustained VT**	17	53.1
**Shock**	9	29.1
**ATP**	15	46.9

Abbreviations: VF, Ventricular Fibrillation; VT, Ventricular Tachycardia; ATP, Anti-Tachycardia Pacing

^*^Two patients had experienced both sustained VT and VF within the last year. The ventricular arrhythmia of seven patients was stopped by both shock and ATP therapies. Five patients had faced with the shock therapy and twelve patients had experienced the ATP therapy more than once within the last year.

In this study, no significant difference was found between the patients with arrhythmia and those without arrhythmia regarding LAV when the parameters were compared with regard to the presence of sustained VT or VF stored by the ICD device. However, LAV index was significantly higher among the patients with arrhythmia. Both groups had similar LVV, LVV index, and EF. Nevertheless, E / E’ ratio was greater whereas septal S and lateral S values were significantly lower among the patients with arrhythmia (Table 4). 

**Table 4.** Comparison of the Parameters with Regard to the Presence of Sustained VT or VF Episodes Stored by the ICD Device within the Last Year[Table-fn fn7186]

	Present (n = 17)	Absent (n = 15)	P
**LAV (mL)**	68 ± 23.39	55.13 ± 20.41	0.100
**LAV index (mL / m^2^) **	39.27 ± 12.19	25.18 ± 7.45	0.004
**LVV (mL)**	182.73 ± 53.5	159.71 ± 59.24	0.280
**LVV index (mL / m^2^)**	95.27 ± 28.38	77.20 ± 30.19	0.170
**LAV / LVV**	42.27 ± 17.30	37.07 ± 13	0.370
**EF (%)**	29.65 ± 4.98	32.6 ± 5.7	0.120
**E / E’**	19.16 ± 10.12	11.7 ± 7	0.030
**Septal S**	4.86 ± 1.65	6.60 ± 1.64	0.010
**Lateral S**	5.54 ± 1.98	7.30 ± 1.56	0.030
**E / A**	1.37 ± 0.99	0.87 ± 0.30	0.090
**Age (years)**	57.18 ± 11.78	59.80 ± 7.6	0.450

Abbreviatins: E / E’, The ratio of the early ventricular filling velocity (E) to early relaxation velocity on tissue Doppler (E’); Septal S, Pulsed-wave tissue Doppler septal peak systolic velocity; Lateral S, Pulsed-wave tissue Doppler letaral peak systolic velocity; E / A, The ratio of the early (E) to late (A) ventricular filling velocities

On the other hand, when the parameters were compared with respect to the shock therapies administered to ventricular arrhythmias by the ICD device, both LAV and LAV index were significantly higher among the patients who had received shock therapy in the last year. However, no significant difference was observed between the two groups concerning LV, LVV index, and EF. The two groups were also similar regarding E / E’ and lateral and septal S waves (Table 5). 

**Table 5.** Comparison of the Parameters with Regard to the Presence of Shock Therapy the ICD Device Had Administered to Ventricular Arrhythmia Episodes within the Last Year

	Present (n = 9)	Absent (n = 21)	P
**LAV (mL)**	73.33 ± 17.64	57.52 ± 23.15	0.040
**LAV index (mL / m^2^) **	40.86 ± 8.47	28.20 ± 11.77	0.010
**LVV (mL)**	185.44 ± 33.84	165.40 ± 64	0.310
**LVV index (mL / m^2^)**	98.57 ± 16.80	80.71 ± 33.73	0.190
**LAV / LVV**	40.33 ± 7.96	39.50 ± 17.90	0.260
**EF (%)**	28 ± 6.04	32.22 ± 4.84	0.080
**E / E’**	18.83 ± 11.18	14.51 ± 8.56	0.260
**Septal S**	4.86 ± 1.34	5.88 ± 1.96	0.180
**Lateral S**	5.88 ± 1.8	6.53 ± 2.1	0.460
**E / A**	1.27 ± 1.06	1.07 ± 0.64	0.550
**Age (years)**	61.78 ± 10.36	57.09 ± 9.73	0.230

The findings of the current study indicated no significant difference between the patients with and those without ATP therapy regarding LAV values. Although LAV index was higher in the patients receiving ATP therapy, the difference was not statistically significant. On the other hand, E / E’ ratio was significantly higher among the patients who had received ATP therapy (Table 6). 

**Table 6.** Comparison of the Parameters with Regard to the Presence of ATP Therapy the ICD Device Had Administered to Ventricular Arrhythmia Episodes within the Last Year

	Present (n = 15)	Absent (n = 17)	P
**LAV (mL)**	63.47 ± 20.52	60.65 ± 24.93	0.550
**LAV index (mL / m^2^)**	35.67 ± 10.90	29.85 ± 12.92	0.230
**LVV (mL)**	190.54 ± 54.23	156.25 ± 55.25	0.100
**LVV index (mL / m^2^)**	100.22 ± 30.12	76.50 ± 26.66	0.060
**LAV / LVV**	37.38 ± 11.92	41.69 ± 17.82	0.840
**EF (%)**	30.40 ± 4.03	31.59 ± 6.53	0.390
**E / E’**	20.33 ± 10.34	12.30 ± 7.28	0.020
**Septal S**	5 ± 1.63	6.27 ± 1.90	0.080
**Lateral S**	5.82 ± 2.04	6.75 ± 1.91	0.280
**E / A**	1.36 ± 1.02	0.97 ± 0.52	0.230
**Age (years)**	56 ± 10.85	60.53 ± 8.91	0.210

In this study, a positive correlation was found between the functional capacity of the patients and LAV (r = 0.52, P = 0.002), LAV index (r = 0.55, P = 0.008), and E / E’ ratio (r = 0.42, P = 0.03). Also, functional capacity was negatively correlated with EF (r = -0.46, P = 0.007).

## 4. Discussion

The present study results showed that LAV and LAV index were correlated with sustained VT frequency within the last 1 year. It was demonstrated that the patients with a greater LAV or LAV index experienced a greater number of ICD shock therapies compared to others within the last year.

Heart failure and serious ventricular arrhythmias are the general findings of LV dysfunction. However, these problems may also arise in the absence of such dysfunction (1). Ventricular arrhythmia and LV dysfunction are independently associated with the mortality risk in the patients with previous myocardial infarction (2-5). Arrhythmic death rates have been found similar in the patients with EF < 30% and > 30% (9). Hence, it is insufficient to determine the treatment strategy based solely on low EF. Supporting this notion, one study showed that low EF could not predict if death resulted from arrhythmia or heart failure, whereas death would most likely be arrhythmic in case of the presence of an inducible tachyarrhythmia, more so particularly if EF > 30% (9).

Diastolic dysfunction is an important predictor of disease severity in the patients with chronic heart failure due to dilated CMP. Also, diastolic dysfunction grade is more closely correlated with prognosis and symptomatic status (10-14).

Diastolic failure is characterized by increased chamber stiffness, increased LV end-diastolic pressures, and limited filling. Changes in myocardial loading conditions which modulate the cardiac electrophysiological properties and are thought to contribute to arrhythmogenesis in the patients with impaired ventricular function and elevated filling pressures (15-18). The cellular mechanisms responsible for this form of mechanoelectrical coupling are generally unknown, but have also been attributed to stretch-activated channels (16) or load-mediated activation of the beta-adrenergic receptors (18).

Previous studies have made it clear that independent risk factors other than EF are present to predict the risk of death, with inducible arrhythmia and diastolic dysfunction being the significant ones. As far as we know, no studies to date have investigated the association between LAV and ventricular arrhythmia frequency in the patients with dilated CMP having previously implanted ICD. Yet, it is necessary to clarify whether there is any relationship between the grade of diastolic dysfunction secondary to systolic dysfunction and the frequency of ventricular arrhythmia.

A previous study (6) found a correlation between LA width and ventricular arrhythmia frequency. However, unlike our study, they used LA diameter instead of LAV and the arrhythmia frequency was determined through a rhythm Holter study. The most serious arrhythmia in that study was non-sustained VT. Our study differs from that study in terms of using LAV and calculation of malignant ventricular arrhythmia frequency within the last year.

In the patients with no previous primary atrial pathology, congenital heart disease, or mitral valve disease, increased LAV typically reflects high filling pressures. During LV, diastole LA is exposed to LV pressure (19). Structural changes in LA may be considered secondary to chronic high filling pressures (8, 20).

The relationship between LAV and diastolic dysfunction resembles that of a random blood glucose level and HBA1c (21). LAV is a marker of chronic diastolic dysfunction. ICD devices may be a guide in diagnosis and treatment of arrhythmias by storing previous arrhythmic events. Similarly, if we assume LA as a memory in which chronic events in LV are stored, we can have an idea about previous diastolic dysfunction. Thus, by reading the events within the last year from both ICD memory and LAV, we tried to elucidate the effect of diastolic dysfunction on ventricular rhythm disorders.

Unlike LA diameter, LAV which is measured through two or three-dimensional echocardiography provides more accurate and reproducible information on LA size when MRI and CT measurements are taken as the reference standards (22-25). Also, this information has a more robust association with cardiovascular outcomes (8, 26, 27).

Body size is a determinant of LA size. Thus, LA size needs to be indexed for body size, and more generally for body surface area (27, 28).

In the current study, 11 subjects were idiopathic dilated CMP patients. Several observational studies have found an increased risk of sudden death in the patients with idiopathic dilated CMP (29-31). Since a significant majority of these deaths are due to ventricular arrhythmias, we included these patients, too.

In our study, LAV values were similar in the patients with and those without malignant ventricular arrhythmia (sustained VT or VF) in the last year. However, LAV index was significantly higher among the patients with arrhythmia (Table 4). Besides, although the mean LAV was higher in the patients with ventricular arrhythmias, the difference was not statistically significant due to the small number of patients. Furthermore, as it is known that LAV is significantly affected by body surface area, it may be suggested that LAV index is more notable. The present study findings revealed no significant difference between the patients with and those without serious ventricular arrhythmic episodes in terms of EF, LVV, and LVV index.

However, both LAV and LAV index were significantly higher in the patients experiencing ICD shock therapy within the last year compared to those with no such therapy (Table 5). Nonetheless, both groups were similar in terms of LVV, LVV index, and EF. Since shock therapies are adjusted to be triggered by fast VT or VF, a more frequent shock therapy in the patients group with a large LA supported our hypothesis that such an increase in LA size leads to more frequent ventricular arrhythmia episodes.

In this study, no significant difference was observed between the patients who had ATP therapy and those who did not regarding LAV. In spite of the fact that LAV index was higher in the ATP therapy group, the difference was not statistically significant. This can be attributed to both small number of patients and, unlike shock therapy, ATP’s wider therapeutic window. In other words, ATP therapy is directed not only against some faster rhythms that impair hemodynamics, but also against various other arrhythmias with different ventricular rates.

The findings of the present study demonstrated that EF, LVV, and LVV index could not differentiate the patients group with a recent ventricular arrhythmia episode from those without. Moreover, it was indicated that among the patients in whom these parameters were similar, LAV and LAV index might be used in detecting those who were at high risk of malignant ventricular arrhythmias.
